# Value of prolonged scrotal drainage after penile prosthesis implantation: a multicenter prospective nonrandomized pilot study

**DOI:** 10.1038/s41443-023-00710-8

**Published:** 2023-05-11

**Authors:** D. Osmonov, A. M. Ragheb, T. Petry, A. Eraky, C. Bettocchi, K. G. Lamers, K. Van Renterghem, M. Tropmann-Frick, E. Chung, K. P. Jünemann, G. Garaffa, H. Porst, A. G. Mohamed, S. K. Wilson

**Affiliations:** 1 Department of Urology and Pediatric Urology, University Hospital Schleswig Holstein, Campus Kiel, Kiel, Germany; 2 Department of Urology, Faculty of Medicine, Beni-Suef University, Beni-Suef, Egypt; 3 Department of Emergency and Organ Transplantation, Urology, Andrology and Kidney Transplantation Unit, University of Bari, Bari, Italy; 4 Departmentof Urology, Jessa Hospital Hasselt, Hasselt, Belgium; 5 Hamburg University of Applied Sciences, Hamburg, Germany; 6 Princess Alexandra Hospital Southside Clinical Unit, Faculty of Medicine, Woolloongabba, QLD Australia; 7 Andrologia Internazionale, Rome, Italy; 8 European Institute for Sexual Health (EIHS), Hamburg, Germany; 9 Instutute of Urologic Excellence, La Quinta, CA USA

**Keywords:** Health care, Medical research, Therapeutics

## Abstract

We aimed to understand the risks and benefits of post-inflatable penile prosthesis (IPP) implantation drainage and optimal duration. Our patients were divided into 3 groups: Group 1 (*n* = 114) had no drain placed, Group 2 had a drain placed for 24 h (*n* = 114) and Group 3 had a drain placed for 72 h (*n* = 117). Postoperative scrotal hematoma and prosthesis infection rates were compared between the groups. The patients from Group 3 demonstrated a statistically significant lower incidence of hematoma on the 10th postoperative day: (*n* = 1, 0.9%) compared to Group 2: (*n* = 11, 9.6%) and Group 1: (*n* = 8, 7%), (*p* = 0.013). However, on the 3rd postoperative day, there was a statistically significant lower incidence of hematoma in both Groups 3 and 2: (0.9% and 6.1%, respectively) vs. Group 1: (11.4%), (*p* = 0.004). Hematoma rates followed the same group order after the first day of surgery: 1.7% (*n* = 2), 5.3% (*n* = 6), and 8.8% (*n* = 10), respectively, (*p* = 0.05). Five patients (4.4%) in Group 1 and four patients (3.5%) in Group 2 developed an IPP associated infection, opposed to only a single patient (0.85%) in Group 3, (*p* = 0.210). We concluded that prolonged scrotal drainage for 72 h after virgin IPP implantation significantly reduces hematoma and infection rates.

## Introduction

To date, inflatable penile prosthesis (IPP) implantation remains the gold standard treatment for erectile dysfunction refractory to medical treatment and is associated with the highest rates of patient satisfaction [1–3]. The last 50 years have been characterized by continuous advances in design and technology of penile implants and by a constant amelioration of surgical techniques and this has significantly improved overall surgical outcomes [4].

One of the most common complications of IPP implantation is postoperative bleeding and hematoma formation [5]. The loose nature and dependency of the scrotal tissues make the scrotum prone to the collection of blood around the pump and in the soft dartoic tissues. A number of measures have been introduced in order to minimize the risk of hematoma formation, spanning from proper patient selection and preparation and meticulous intraoperative hemostasis to adequate postoperative management. Postoperative measures include close suction drainage and the application of a compressive dressing such as the Mummy Wrap on the genitalia. To date, the decision on whether to leave a drain or not remains controversial [6–8]. The arguments in favor of close drain insertion include the lower risk of hematoma formation, which translates into a quicker and less uncomfortable recovery for the patient and an earlier activation of the device [9, 10].

Surgeons in favor of close drain insertion claim that this practice is not associated with an increased risk of infection [7, 11–13]. On the other hand, opponents regard drain insertion as a potential source of infection [14].

Our aim was to add to the body of evidence supporting the benefit of IPP implantation drainage as well suggest the optimal duration of drainage. Until an adequately designed prospective randomized controlled study is conducted to assess the potential benefits of inserting a drain during implantation of IPP, the current series suggests that closed drainage insertion after IPP implantation is associated with a significant reduction of hematoma and infection rates.

## Methods

We conducted a multicenter prospective nonrandomized pilot study. Surgery was carried out by four European and one Australian high-volume surgeons at five different centers of excellence. Only virgin IPP cases were included in the series. Patients with Peyronie’s disease or severe fibrosis after priapism were not included. Patients on prophylactic anticoagulation were bridged prior to surgery. The patients that could not be bridged were excluded. All procedures were performed through the penoscrotal access and following the same surgical steps according to the Kiel protocol for IPP implantation. For stay sutures, overlapping stay stitches were utilized to achieve water tight closure of the corporotomies after implant placement. Our drain of choice was a 12 French closed suction drain routing below the corporotomies and behind the pump in all the patients. For closure, Dartos and skin layers were closed by interrupted sutures in a locking fashion for wound sealing. All patients received the same perioperative antibiotic prophylaxis. Devices were left partially inflated (75%) and a mummy wrap was applied for all the patients for 24 h. The urethral catheter was removed after 48 h and all patients were discharged after the 3rd postoperative day. Patients were divided into 3 groups. No drain was inserted in Group 1 patients (*n* = 114); a drain was inserted for 24 h in Group 2 (*n* = 114) and for 72 h in Group 3 (*n* = 117). Out of the surgical outcomes the presence or absence of postoperative scrotal hematoma and infection were the focus of our study. They were evaluated and compared between the three groups. We defined hematoma as a scrotal swelling correlated with ultrasonographic evidence of scrotal free-floating fluid. An US scan was carried out on day 1, 3 and 10 postoperatively. Patients were followed up for 80 days postoperatively. This study was approved by the ethical committee of the Christian Albrecht’s University of Kiel, Germany (D 444/19).

### Statistical analysis

Using SPSS version 26 for windows, data was entered, coded and analyzed. All variables were categorized where they were described as numerical and percentage. Chi squared and Exact tests were used when required (referred in the tables). Binary logistic regression analysis was used to compare the prevalence of complications in the 3 different Groups. A *p* value < 0.05 was considered significant.

## Results

**Fig. 1:**
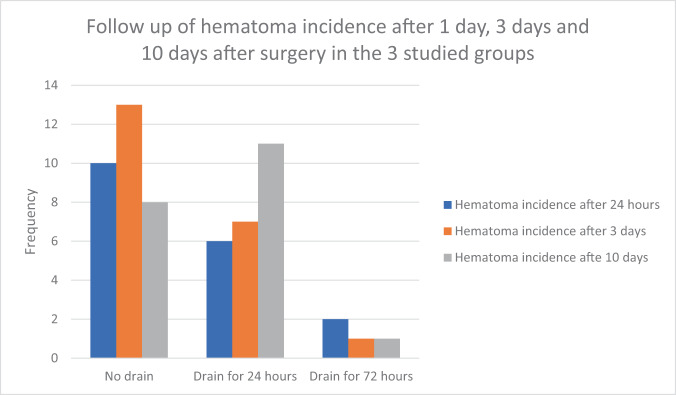
Frequency of hematoma among the studied groups. Hematoma rates were highest in the no-drain group 24 h after surgery [8.8%], followed by the 24 h group [5.3%] and lowest in the 72 h group [1.7%] (*p* = 0.05). Although hematoma rates failed to demonstrate a statistical difference on the 3rd postoperative day comparing the no-drain to the 24 h drain group [11.4% and 6.1%, respectively] (*p* = 0.157) and the 24 h to the 72 h, separately [6.1% and 0.9 %, respectively] (*p* = 0.031), the hematoma rates were significantly different comparing the 2 drained groups vs. the no-drain group (*p* = 0.004). On the 10th day post-surgery, we identified a statistically significant lower incidence of hematoma with prolonged drainage [0.9%] compared to short term drainage [9.6%] and non-drainage [7%], (*p* = 0.013).

Table 1 demonstrates that there was no statistically significant difference among the 3 groups with regards to age, BMI, comorbidities, type of implant utilized and operative time. (Table 1) After the first day of surgery, the no-drain group ranked highest in hematoma incidence [8.8%], followed by the 24 h group [5.3%] and the 72 h group [1.7%] (*p* = 0.05). On the 3rd postoperative day, both drained groups (24 and 72 h drain) demonstrated a statistically significant lower incidence of hematoma in [6.1% and 0.9%, respectively] vs. the no-drain group [11.4%] (*p* = 0.004). Nevertheless, when we separately compared the no-drain group to the 24 h drain group (*p* = 0.157) and the 24 h to the 72 h drain group (*p* = 0.031), this difference, although may be clinically significant, wasn’t statistically significant. It was statistically significant only when we compared the 72 h group with the no-drain group (*p* = 0.001). On the 10th day after surgery, we identified a statistically significant lower incidence of hematoma in patients who had a drain for 72 h [0.9%] compared to the 24 h group [9.6%] and the no-drain group [7%], (*p* = 0.013). There was also a statistically significant lower incidence of hematoma when we separately compared patients in the no-drain group with the 24-group (*p* = 0.017) and patients in the 24 h group with 72 h group (*p* = 0.003). However, the difference wasn’t statistically significant when we compared the no-drain group with the 24 h group (*p* = 0.477). (Table 2 and Fig. 1).

**Table 1:** Comparison between the studied groups regarding their baseline characteristics and intraoperative time.

Parameters	No drain (no = 114)	24 h drain (no = 114)	72 h drain (no = 117)	*P* value
Age				
30–49	7 (6.1%)	7 (6.1%)	11 (9.4%)	0.791
50–69	67 (58.8%)	66 (57.9%)	70 (59.8%)	
≥70	40 (35.1%)	41 (36.0%)	36 (30.8%)	
BMI				
≤24.9	23 (20.2%)	26 (22.8%)	28 (23.9%)	0.161
25–29.9	57 (50.0%)	40 (35.1%)	53 (45.3%)	
≥30	34 (29.8%)	48 (42.1%)	36 (30.8%)	
Peyronie’s disease	25 (21.9%)	23 (20.2%)	19 (16.2%)	0.533
Diabetes				
No	82 (71.9%)	89 (78.1%)	82 (70.1%)	0.434
Type I	5 (4.4%)	6 (5.3%)	10 (8.5%)	
Type II	27 (23.7%)	19 (16.7%)	25 (21.4%)	
Peripheral vascular disease	81 (71.1%)	79 (69.3%)	83 (70.9%)	0.948
Cystectomy	4 (3.5%)	0 (0.0%)	3 (2.6%)	0.151 (ET)
Radiation therapy	3 (2.6%)	6 (5.3%)	4 (3.4%)	0.605
Radical prostatectomy	8 (7.0%)	12 (10.5%)	14 (12.0%)	0.432
previous urinary infections	11 (9.6%)	8 (7.0%)	5 (4.3%)	0.275
Type of the implant	24 (21.1%)			
AMS 700®	2 (1.8%)	21 (18.4%)		
ZS*I®*	88 (77.2%)	0 (0.0%)	22 (18.8%)	0.863
Coloplast OTR®		93 (81.6%)	0 (0.0%)	0.217
			95 (81.2%)	0.656
Surgical time				
>60 min	10 (8.8%)	5 (4.4%)	5 (4.3%)	0.252
≤60 min	104 (91.2%)	109 (95.6%)	112 (95.7%)	

*BMI* Body mass index, *AMS* American Medical Systems, *ZSI* Zephyr Surgical Implants, *OTR* One touch release.

**Table 2:** Incidence of hematoma/infection among the studied groups.

Parameters	No drain (no = 114)	24 h drain (no = 114)	72 h drain (no = 117)	*P* value	Statistical test
Evidence of hematoma by US after 24 h of the operation	10 (8.8%)	6 (5.3%)	2 (1.7%)	0.054	Chi-Squared
Hematoma after 24 h in:					Chi-Squared
>60 min	4/10 (40.0%)	4/5 (80.0%)	1/5 (20.0%)	0.147	
≤60 min	6/104 (5.8%)	2/109 (1.8%)	1/112 (0.9%)	0.097	
Evidence of hematoma by US after 3 days of the operation	13 (11.4%)	7 (6.1%)	**1 (0.9%)**	P1 = 0.157 P2 = 0.001^a^ P3 = 0.031^a^	Chi-Squared
Hematoma after 3 days in:					Chi-Squared
>60 min	5/10 (50.0%)ab	5/5 (100.0%)	**0/5 (0.0%)**	>60 min P1 = 0.061 P2 = 0.060 P3 = 0.003^a^ ≤60 min P1 = 0.042^a^ P2 = 0.012^a^ P3 = 0.562	
≤60 min	8/104 (7.7%)a	2/109 (1.8%)	**1/112(0.9%)**		
Evidence of hematoma by US after 10 days of the operation	8 (7.0%)	11 (9.6%)	**1 (0.9%)**	0.013^a^ P1 = 0.477 P2 = 0.017^a^ P3 = 0.003^a^	Chi-Squared
Hematoma after 10 days in:					Chi-Squared
>60 min	4/10 (40.0%)	3/5 (60.0%)	**0/5 (0.0%)**	0.157 ≤60 min P1 = 0.283 P2 = 0.146 P3 = 0.016^a^	
≤60 min	4/104 (3.9%)	8/109 (7.3%)	**1/112 (0.9%)**		
IPP infection	5 (4.4%)	4 (3.5%)	1 (0.9%)	0.210	Chi-Squared
IPP infection 3 months	3 (2.6%)	6 (5.3%)	2 (1.7%)	0.296	Chi-Squared
IPP explantation due mechanical failure of device within 3 month					Chi-Squared
No	110 (96.5%)	98 (86.0%)	108 (92.3%)	P1 = 0.004^a^ P2 = 0.155 P3 = 0.123	
Reroute	4 (3.5%)	16 (14.0%)	9 (7.7%)		
Wilsons Modeling	16 (14.0%)	3 (2.6%)	3 (2.6%)	P1 = 0.002^a^ P2 = 0.002^a^ P3 > 0.999	Chi-Squared

P1 (no drain vs. 24 h drain).

P2 (no drain vs. 72 h drain).

P3 (24 h drain vs. 72 h drain).

*US* Ultrasound, *IPP* Inflatable penile prosthesis.

^a^*P* value is significant. Different letters denote significant difference between groups.

The stratification of the groups by surgical time (under or above 60 min) did not affect our results regarding the incidence of hematoma with superiority remaining for the 72 h drain group.

Early postoperative infection rates were highest in the no drain group [4.4%] followed by the 24-drain group [3.5%] and lowest in the 72 h group [0.9%]. Despite the clinical significance of such differences, there were no significant statistical differences (*p* = 0.210). Interestingly, the only significant factor associated with the increased incidence of postoperative infection was the occurrence of a hematoma at 24 h after surgery (*p* = 0.01). The presence of a drain was not found to be a risk for infection. This was demonstrated after adjusting for age, presence of diabetes and intraoperative time, in the presence of a drain for 24 and 72 h, and the presence of hematoma at 24 h (Table 3).

**Table 3:** Binary logistic regression analysis for prediction of factors associated with the increase in the incidence of postoperative infection.

Predictors	*P* value	OR	95% C.I. for EXP OR	
			Lower	Upper
*Groups*				
No drain	Reference	Reference	Reference	Reference
Drain for 24 h	0.848	1.173	0.230	5.991
Drain for 72 h	0.391	0.354	0.033	3.793
*Age*				
Age [30–49] years	Reference	Reference	Reference	Reference
Age 50–69	0.586	0.558	0.068	4.550
Age ≥70	0.261	0.258	0.024	2.734
Presence of DM	0.134	3.324	0.691	15.992
Surgical time >60 min	0.071	5.038	0.873	29.094
Hematoma at 24 h	0.001	16.317	3.020	88.160
Model summary	*R*^2^ = 0.387 *X*^2^ = 32.3 at df(7) *P* value < 0.001			

## Discussion

Minimizing hematoma formation after IPP insertion is paramount to reduce patient discomfort, to allow for a quicker healing and activation of the device, and possibly, to reduce the risk of infection of the device. In a series of 917 patients, Wilson et Al. assessed the efficacy of closed drain insertion and compressive dressing at preventing the formation of scrotal hematomas. In particular, he subdivided his patients in 3 groups. In the first group (163 patients) only a pressure dressing to the genitalia was applied while in the second group (255 patients) a drainage and pressure dressing were used. In the third group instead (555 patients) a combination of insertion of a drain, application and a compressive dressing and partial inflation of the cylinders (70% inflation) were attempted with the aim of reducing hematoma formation. The overall risk of hematoma formation, which was the highest in group 1, was 3.6% in the second group and 0.9% in the third group [8].

In another series of 600 patients where CSD was placed for 24 h with partial inflation of the devices without any pressure dressing, the reported prevalence of delayed hematoma was 0.5% (>5 days postoperative). According to Garber et al., hematoma formation was related to premature administration of anticoagulants or early vigorous physical activity [15].

Apoj et al. used a combination of pressure dressing, full inflation of devices and drainage placement for 24 h. The authors reported no incidence of hematoma or infection in 169 IPP undergoing patients [16].

Sadeghi-Nejad et al. evaluated the rate of infection and hematoma formation using closed suction drain in 425 patients after IPP implantation through a penoscrotal approach. Nonantibiotic impregnated non-hydrophilic devices were used in this study. The rate of infection was 3.3%, while the rate of hematoma was 0.7%. Drainage was removed after the first 24 h. They concluded that the placement of a closed suction drain did not increase the rate of infection and was associated with a lower incidence of hematoma formation [7].

The hypothetical relation between the utilization of a scrotal drain and IPP infection is questionable, although the theoretical risk of retrograde migration of organisms from the point of insertion of the drain to the scrotal cavity may be considered. In order to shed more light on this concept, Wallen assessed cultures obtained from the proximal and distal end of 130 drains placed for 48 or 72 h after IPP surgeries. Only 1.5% grew bacteria, but none of them developed a clinical infection. The rate of hematoma formation was 1.5% in the same study [17]. Similar results were reported by Rojas-Cruz et Al. in a series of 63 patients were, although drain cultures were positive in 6% of patients none of these patients developed a clinical infection. The rate of hematoma formation was 1.5%. Based on these findings, they concluded that there was no relation between using CSD and IPP infections [18].

Meng et al. compared brief (<24 h) vs. prolonged (average duration 4.7 days) closed suction drain insertion after IPP surgery. They failed to identify any significant difference in infection rates between the two groups (0.9% vs. 1.7%, respectively, *p* = 1), although hematoma formation rate was significantly lower in the prolonged drainage group 14.4% vs. 26% (*p* = 0.02). In this series infection rates did not increase in the prolonged drainage group suggesting that the retrograde migration of organisms theory was unlikely [19].

In a multicenter prospective study (PROPPER study), 1348 patients were stratified into drain 634 (47%), and no-drain 714 (53%) groups. The study group observed hematoma formation only in the drain group (0.006%) while none in the no-drain group (*p* = 0.034) possibly because drains were more likely used in the more complex (and more likely to bleed) cases. Even in this series drainage usage was not associated with higher infection rates [16].

In our study the surgical outcome in patients post IPP without drainage (Group 1- 114 patients), with short term drainage (Group 2- 114 patients, 24 h) and prolonged drainage (Group 3- 117 patient, 72 h) were evaluated. On the tenth follow up day, the prevalence of hematoma was the lowest in Group 3 [0.9%] and highest in Group 1 [9.6%], (*p* = 0.013). The incidence of infection requiring device removal was highest in Group 1 [4.4%], followed by Group 2 [3.5%] and lowest in Group 3 [0.9%] (*p* = 0.210). The findings of the current series support our previous conclusion that prolonged drainage reduces the risk of hematoma formation without increasing the risk of infection [20].

We are aware of the strengths and limitations in our study. Although we studied a large cohort yet population distribution was not randomized. Preoperative counseling and postoperative management of the patients may be heterogeneous owing to the multicentric nature of our study.

## Conclusions

Prolonged drainage for up to 72 h after virgin IPP implantation significantly reduces hematoma formation and the risk of infections regardless of surgical time.

Due to the nature of this series, its results need to be confirmed by larger randomized trials, which can be possible only after the establishment of the European Registry.

## Data Availability

The dataset on which this paper is based is securely retained with the corresponding author. Documentation and methods used to support this study are available via the corresponding author.
